# Variable roles of interleukin-17F in different cancers

**DOI:** 10.1186/s12885-021-08969-0

**Published:** 2022-01-11

**Authors:** Tiina Mikkola, Rabeia Almahmoudi, Tuula Salo, Ahmed Al-Samadi

**Affiliations:** 1 Department of Oral and Maxillofacial Diseases, University of Helsinki, Clinicum, Biomedicum Helsinki 1, C223b, P.O. Box 63 (Haartmaninkatu 8), 00014, Helsinki, Finland; 2 Translational Immunology Research Programme (TRIMM), University of Helsinki, Helsinki, Finland; 3 Cancer and Translational Medicine Research Unit, University of Oulu, Oulu, Finland; 4 Medical Research Centre, Oulu University Hospital, Oulu, Finland; 5 HUS, Helsinki University Hospital, Helsinki, Finland

**Keywords:** IL-17F, cancer, Systematic review, Lymphocytes, Prognostic, Polymorphism

## Abstract

**Background:**

Interleukin (IL)-17 family is a group of six cytokines that plays a central role in inflammatory processes and participates in cancer progression. Interleukin-17A has been shown to have mainly a protumorigenic role, but the other members of the IL-17 family, including IL-17F, have received less attention.

**Methods:**

We applied systematic review guidelines to study the role of IL-17F, protein and mRNA expression, polymorphisms, and functions, in cancer. We carried out a systematic search in PubMed, Ovid Medline, Scopus, and Cochrane libraries, yielding 79 articles that met the inclusion criteria.

**Results:**

The findings indicated that IL-17F has both anti- and protumorigenic roles, which depend on cancer type and the molecular form and location of IL-17F. As an example, the presence of IL-17F protein in tumor tissue and patient serum has a protective role in oral and pancreatic cancers, whereas it is protumorigenic in prostate and bladder cancers. These effects are proposed to be based on multiple mechanisms, such as inhibition of angiogenesis, vasculogenic mimicry and cancer cell proliferation, migration and invasion, and aggravating the inflammatory process. No solid evidence emerged for the correlation between IL-17F polymorphisms and cancer incidence or patients’ prognosis.

**Conclusion:**

IL-17F is a multifaceted cytokine. There is a clear demand for more well-designed studies of IL-17F to elucidate its molecular mechanisms in different types of cancer. The studies presented in this article examined a variety of different designs, study populations and primary/secondary outcomes, which unfortunately reduces the value of direct interstudy comparisons.

## Introduction

Cancer caused almost 9.6 million deaths globally in 2018 [1]. The incidence of cancers can be decreased to some extent by avoiding known risk factors, such as tobacco smoking and alcohol consumption [2], but several unknown environmental, genetic, and epigenetic reasons likely exist for cancer progression. Thus, safer and more effective treatments are continuously being sought for different cancer types.

Cancer biomarkers are biological compounds detectable in tissues or body fluids. They are applied for the prognosis of various cancers and to predict the outcome and efficiency of treatments [3]. Cytokines, one group of biomarkers, are proteins that participate in cell signaling and mediate innate and adaptive immune system responses. Some cytokines, such as interleukin (IL)-2 and IL-15, seem to also be relevant in cancer immunotherapy [4].

Interleukin (IL)-17F is a member of the IL-17 cytokine family, which contains six members (IL-17A-F). IL-17F is produced by several cells, including activated CD4^+^ T cells, monocytes, basophils, and mast cells [5]. IL-17A and IL-17F share the greatest homology with each other, and they have two common receptors: IL-17RA and IL-17RC. IL-17A was the first IL-17 cytokine identified, and it plays important roles in host defense, inflammation, allograft rejection, and autoimmune diseases such as psoriasis [6–8]. IL-17A has mainly been reported as a protumorigenic factor, however some studies showed it as an antitumorigenic cytokine [6, 9]. Other members, including IL-17F, have been far less extensively studied, also in cancer [10].

Here, we systematically collected literature concerning the expression, polymorphisms, and function of IL-17F in various cancers and reviewed the role of IL-17F as a pro- or antitumorigenic molecule. We also analysed data related to the proposed molecular mechanisms by which IL-17F affects cancer development and progression.

## Methods

This review study was registered at the international prospective register of systematic reviews PROSPERO under registration number CRD42020186465.

We followed the Preferred Reporting Items for Systematic Reviews and Meta-Analyses (PRISMA) guidelines and conducted a search using PubMed, Ovid Medline, Scopus, and Cochrane Library databases. We searched the terms interleukin-17F (interleukin 17f OR il 17f OR interleukin-17f OR il-17f) and cancer (cancer OR neoplasm* OR carcinoma* OR malignan* OR tumo?r* OR sarcoma* OR leukemia* OR lymphoma* OR adenocarcinoma*), with an asterisk (*) indicating truncation and a question mark (?) indicating wildcard characters, from titles, abstracts, and keywords. We conducted two searches, the first one was on the 15th of May 2020, and the second search covered the period from 16th of May 2020 until 9th of October 2021.

**Fig. 1:**
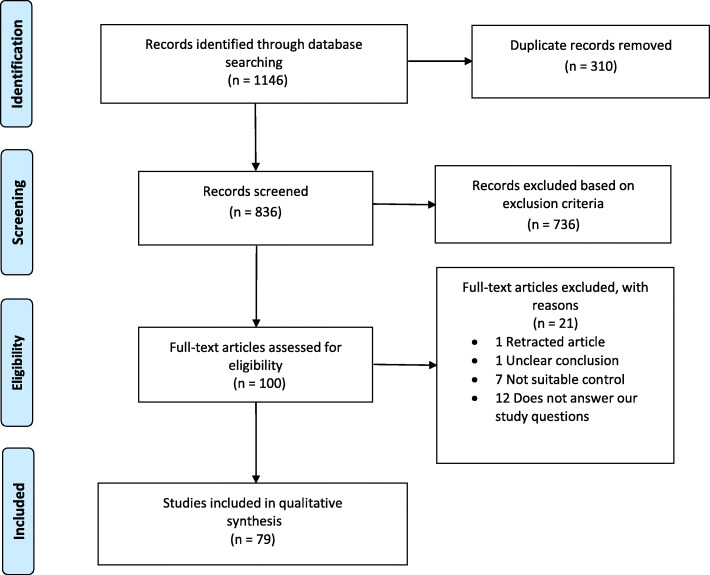
Flow chart of the search strategy and the studies included and excluded at various steps

We identified a total of 1146 records through database searching and removed 310 duplicates, leaving 836 records for screening. Three researchers (R.A. and T.M. or A.A.) screened the records independently and were blinded to each other’s decisions. Disagreements in included and excluded articles were resolved by reaching a consensus between the researchers. We excluded 736 records because they were reviews, case reports, letters, book chapters, conference abstracts, written in languages other than English, or irrelevant to our review. In addition, we found a few more duplicates during manual record screening. We assessed 100 full-text articles for eligibility, excluded a further 21, and thus, included 79 articles in this review (Fig. 1). We recorded all data extraction results using Excel.

We extracted all data concerning cancer type, study size, methodology, and main findings with *p*-values from the articles. We included study population in the extraction of IL-17F polymorphism studies.

## Results

### Protein and mRNA expression of IL-17F in various cancers

We present the studies on IL-17F mRNA and protein expression levels in Table 1 based on cancer type listed alphabetically. Below, we describe the results in the order of the most studied cancers of each category.

**Table 1:** Interleukin-17F expression in cancer

Cancer	Sample and cancer type	Target	Method	Patients + control	Main findings	Authors
Bladder	Surgical resection	Protein	IHC	80 (bladder cancer) + 23 (cystitis) + 6 (hyperplastic bladder polyps)	IL-17F was overexpressed in the bladder cancer group (*p* < 0.01)	Liu et al. 2016 [11]
Breast	Blood	Protein	Multiplex	150 + 60	Soluble levels of IL-17F were similar between breast cancer patients and healthy control (data not shown)	Avalos-Navarro et al. 2019 [12]
	Surgical specimens	Protein	IHC	180	IL-17F was not associated with pCR rate but tumors with IL17F infiltrates were significantly smaller than those without them (*P* = 0.041).	Oda et al. 2012 [13]
Colorectal	Cultured rectal cancer biopsy	Protein	Multiplex ELISA	12 (rectal cancer) + 8 (normal)	IL-17A/F was secreted at significantly higher levels from the rectal cancer secretome than the normal rectal secretome (*p* = 0.003).	Heeran et al. 2020 [14]
	CRC, surgical resection	Protein, mRNA	RT-qPCR, WB, IHC	67	IL-17F was overexpressed in CRC tumor tissues compared with paired non-tumor mucosa at both mRNA and protein levels. Overexpression of IL-17F was associated with worse RFS (*p* = 0.03) and OS (*p* < 0.05)	Chen et al., 2019 [15]
	CRC, endoscopic biopsy	Protein	IHC	29 (CRC) + 17 (UC) + 7 (CHP)	Positive IL-17F was significantly higher in UC and polyp samples compared with CRC (*p* = 0.00001 and *p* = 0.0007)	Liu et al. 2018 [16]
	CRC, serum	Protein	Multiplex	122	No association between IL-17F and OS/PFS	Lereclus et al. 2017 [17]
	CC, surgical resection, serum	Protein	Bio-Plex	33	Elevated levels of IL-17F were associated with advanced disease. Stage IV showed elevated systemic levels of IL-17F compared to stages I-III (p all< 0.05).	Sharp et al. 2017 [18]
	CRC, serum	Protein	ELISA	109 + 52	No IL-17F was detected in patients’ sera and only one healthy individual had IL-17F in his serum.	Nemati et al. 2015 [19]
	CRC, cultured surgical resection	Protein	IHC/IF	10 + 10	IL-17F was decreased in CRC compared to healthy control	Al-Samadi et al. 2015
	CC, surgical resection	Protein + mRNA	IHC, WB, RT-qPCR	40	Lower levels of IL-17F mRNA were found in cancer tissue compared to normal mucosa (p < 0.05)	Tong et al. 2012 [20]
Oral	Oral and/or oropharyngeal cancer, saliva	protein	ELISA	71	The higher salivary concentrations of IL-17F was significantly associated with disease advancement.	Zielińska et al. 2020
	OTSCC, surgical resection	Protein	IHC/IF	83	Extracellular IL-17F at the tumor invasion front was associated with better disease-specific survival in patients with all-stages and early-stages of oral tongue SCC. (*p* = 0.001)	Almahmoudi et al. 2019 [21]
	OSCC, blood	Protein	ELISA	58 + 52	IL-17F was decreased in OSCC patient samples compared to healthy control (p < 0.05).	Xiaonan et al. 2019 [22]
	OSCC, serum	Protein	ELISA	85 (OSCC) + 15 (leukoplakia) + 28 (healthy)	IL-17F was decreased in OSCC patient samples compared to healthy controls (p < 0.05). Patients with smoking habit had higher IL-17F.	Ding et al. 2015 [23]
Leukemia	CLL, blood	Protein	FCM	21 + 9	No significant association between TH17F cells and CLL	Sherry et al. 2015 [24]
	B-CLL, serum and cell lysates	Protein	WB, ELISA	23 + 13	IL-17F is less expressed in PMNs and B-lymphocytes of patients compared to cells of healthy subjects. IL-17F was significantly increased in serum of stage IV disease patients compared with healthy subjects and stage 0/I and III patients.	Garley et al., 2014 [25]
Liver	HCC, blood	Protein	Bio-Plex	87 + 87	IL-17F levels not associated with HCC	Shen et al. 2018 [26]
	HCV-HCC, surgical resection	mRNA	qRT-PCR	40 (cancerous + adjacent non-cancerous tissues)	IL-17F positive frequency was higher in cancerous tissue than in non-cancerous tissue	Wu et al. 2017 [27]
Lung	surgical resection	Protein	IHC	55 + 12	Expression of IL-17F was positively associated with tumor differentiation and negatively associated with lymph node metastasis and TNM staging (p all< 0.05)	Li et al. 2019 [28]
	NSCLC, surgical resection	Protein	IHC	29 (squamous cell carcinoma) + 30 (adenocarcinoma) + 10 (healthy control)	IL-17F immunoreactivity was increased in both SCC and ADC tissue compared with healthy control (p < 0.05). IL-17F immunoreactivity was expressed principally in macrophages, but also epithelial cells and some malignant cells.	Huang et al., 2018 [29]
	NSCLC, serum	Protein	Multiplex	50 + 14	IL-17F was decreased in squamous cell carcinoma stage M1 compared to M0 (P < 0.05) and in stages IIIB-IV compared to stages I-IIIa (p < 0.01)	Yang et al. 2015 [30]
Lymphoma	BIA-ALCL, surgical resection	Protein	IHC	4 (BIA-ALCL) + 4 (healthy) + 10 (LP) + 6 (pcALCL)	IL-17F expression was weaker in BIA-ALCL tumor cells	Kadin et al. 2016 [31]
	AIDS-NHL, serum	Protein + mRNA	multiplex immunoassay	176 (AIDS-NHL) + 176 (HIV+)	No significant association between IL-17F expression and AIDS-NHL	Vendrame et al. 2014 [32]
	CTCL, skin biopsy	mRNA	PCR	60	IL-17F was expressed more in progressive CTCL compared to non-progressive disease (p < 0.05)	Willerslev-Olsen et al. 2014 [33]
	CTCL, surgical resection	mRNA	PCR	60	IL-17F^+^ patients had a significantly increased risk of disease progression (odds ratio = 2.75; *p* = 0.025) compared with IL-17F- patients	Krejsgaard et al. 2013 [34]
	CTCL, surgical resection	mRNA	RT-qPCR	21 (CTCL) + 5 (psoriasis) + 6 (normal)	IL17F mRNA levels were not significantly elevated in lesional skin of CTCL	Miyagaki et al. 2011 [35]
	CTCL, surgical resection	mRNA	RT-PCR	62	IL-17F genes were expressed in poor prognosis clusters, and seemed to strongly correlate with an advanced and/or progressive disease	Litvinov et al. 2010 [36]
Ovarian	surgical resection	Protein	FCM	24 (cystadenocarcinoma) + 25 (cystadenoma) + 11 (serous borderline tumors) + 20 (control)	Number of IL-17F-positive TH17 cells is not increased or decreased in ovarian cancer	Winkler et al. 2017 [37]
	ascites	Protein	Cytokine profiling kit	266	No association between IL-17F and OS	Chen et al. 2015 [38]
Pancreas	serum	Protein	Luminex	78 (pancreatic adenocarcinoma) + 41 (control) + 40 (chronic pancreatitis) + 20 (recurrent acute pancreatitis)	IL-17F was decreased in pancreatic adenocarcinoma when compared with chronic pancreatitis (*p* = 0.023)	Park et al. 2020 [39]
Prostate	surgical resection	Protein	IHC	116 (prostate cancer) + 10 (BPH)	A significant higher IL-17F expression was found in the prostate cancer group in comparison to the BPH group (p < 0.05).	Janiczek et al. 2020 [40]
	surgical resection	Protein	IHC	29 (prostate adenocarcinoma) + 47 (BPH) + 6 (control)	IL-17F was elevated in BPH (*p* = 0.014) and prostate adenocarcinoma (*p* = 0.026) compared to healthy controls.	Liu et al. 2015 [41]
Skin	Skin BCC, serum	Protein	ELISA	81 + 53	IL-17F levels not associated with cancer risk	Mohammadipour et al. 2019 [42]

Abbreviations: AIDS-NHL = HIV-infection associated non-hodgkin lymphoma, ADC = adenocarcinoma B-CLL = B-cell chronic lymphocytic leukemia, BCC = basal cell carcinoma, BIA-ACLC = breast implant-associated anaplastic large cell lymphoma, BPH = bening prostatic hyperplasia, CC = colon cancer, CHP = colorectal hyperplastic polyps, CLL = chronic lymphocytic leukemia, CRC = colorectal cancer, CTCL = cutaneous T-cell lymphoma, ELISA = enzyme-linked immunosorbent assay, FCM = flow cytometry, HCC = hepatocellular carcinoma, HCV-HCC = hepatitis C virus-associated hepatocellular carcinoma IF = immunofluorescence, IHC = immunohistochemistry, LP = lymphomatoid papulosis, NSCLC = non-small cell lung cancer, OS = overall survival, OSCC = oral squamous cell carcinoma, OTSCC = oral tongue squamous cell carcinoma, pcALCL = primary cutaneous anaplastic large cell lymphoma, pCR = pathological complete response, PCR = polymerase chain reaction, PFS = progression-free survival, PMNs = polymorphonuclear cells, RFS = relapse-free survival, RT-PCR = real-time or reverse transcription polymerase chain reaction, RT-qPCR = quantitative real-time or reverse transcription polymerase chain reaction, SCC = squamous cell carcinoma, UC = ulcerative colitis, WB = western blotting

#### IL-17F expression is linked to colorectal cancer

The expression of IL-17F has been investigated most in colorectal cancer (CRC) [14–20, 43]. IL-17F expression in CRC tissue samples was studied in 4 articles [15, 16, 20, 43]. In three articles, IL-17F expression in tumour sections was decreased [16, 20, 43]. Al-Samadi et al. [43] showed by immunohistochemistry that IL-17F level was decreased in CRC compared with healthy controls, and similarly, Liu et al. [16] found less IL-17F in CRC than in ulcerative colitis or polyp samples. In addition, IL-17F mRNA expression was reduced in colon cancer according to Tong et al. [20]. However, Chen et al. [15] reported recently that IL-17F was overexpressed in tumour mucosa compared with paired non-tumour mucosa.

Serum levels of IL-17F in CRC patients were studied in 3 articles [17–19]. In one publication, elevated serum, together with conditioned media from cultured surgical resection, levels of IL-17F were associated with advanced colon cancers [18], whereas no association between serum levels and overall survival or progression-free survival among CRC patients was detected in another publication [17], and in a third publication, no detectable IL-17F levels in CRC patients’ serum were found [19].

Heeran et al. [14] showed that IL-17A/F secretion from the cultured rectal cancer biopsy was significantly higher than the normal rectal tissue, however the study did not report the level of IL-17F alone.

#### IL-17F has mainly a protective role in oral squamous cell carcinoma, but not in skin basal cell carcinoma

Four articles investigated the expression of IL-17F in oral cancers [22, 23, 44, 45]. Three articles reported a protective role of IL-17F in oral cancer while the fourth one suggested a protumorgenic effect for IL-17F. Extracellular IL-17F at the tumour invasion front was associated with better disease-specific survival among oral tongue squamous cell carcinoma (OTSCC) patients [44]. In two studies, serum-derived IL-17F was decreased in OSCC patients compared with healthy controls [22, 23]. On the other hand, the concentration of IL-17F in the saliva of oral and oropharyngeal cancer patients was significantly associated with disease progression [45]. To conclude, IL-17F protein in tissue and serum, but not in saliva, seems to possess an antitumorigenic role in oral cancers. By contrast, in skin basal cell carcinoma, serum levels of IL-17F were not associated with cancer risk [42].

#### Variation in IL-17F expression in lymphomas and leukemia

In six articles, the expression of IL-17F in lymphomas was evaluated [31–36]. Kadin et al. [31] found that IL-17F expression was weaker in breast implant-associated anaplastic large cell lymphoma (BIA-ALCL) cells than in benign capsular infiltrates. In three articles, tumoral IL-17F mRNA expression was linked to progressive cutaneous T-cell lymphoma (CTCL) [33, 34, 36], which indicates a protumorigenic role of IL-17F in CTCL. However, Miyagaki et al. [35] did not find elevated levels of IL-17F mRNA in CTCL, and no association between IL-17F serum level and risk for HIV-associated Non-Hodgkin B-cell lymphoma was detected by Vendrame et al. [32].

Two studies addressed the role of IL-17F in chronic lymphocytic leukaemia (CLL) [24, 25]. The first publication showed an increase in serum IL-17F in stage IV B-CLL patients compared with healthy controls and stage 0/I and III patients [25]. The same study found a lower IL-17F expression in PMNs and B-lymphocytes of patients compared to cells of healthy subjects [25]. The second study reported no significant association between TH17F^+^ cells and CLL [24].

#### No consensus on IL-17F levels in lung cancer

Regarding lung malignancies, we found three studies in which IL-17F was measured [28–30]. Huang et al. [29] documented that IL-17F immunoreactivity was increased in both squamous cell carcinoma and adenocarcinoma tissues compared with healthy controls. In contrast, according to Li et al. [28], IL-17F was positively associated with tumour differentiation and negatively with lymph node metastasis and TNM staging. Similarly, Yang et al. [30] found that IL-17F, in the patients’ serum, was decreased in more progressive disease.

#### Amount of IL-17F varies in breast, ovarian, and prostate cancers

Two studies have analysed IL-17F protein amount in breast cancer patients [12, 13]. Oda et al. [13] noted that IL-17F^+^ tumour infiltrate T-cells were associated with smaller tumour size. Avalos-Navarro et al. [12] did not find an association between serum IL-17F expression and breast cancer. In ovarian cancer, levels of IL-17F^+^ Th17-cells were similar between cancer and control groups [37], and IL-17F in the ascites fluid was not associated with patients’ overall survival [38]. As for prostate cancer, two studies reported that IL-17F was overexpressed in prostate cancer samples relative to healthy controls [41] or benign prostatic hyperplasia [40].

#### Expression of IL-17F in liver, pancreatic, and bladder cancers varies

In liver cancer, one study reported no association between IL-17F protein levels and hepatocellular carcinoma [26], whereas another one suggested that IL-17F mRNA was more often present in cancerous than in adjacent non-cancerous tissue of hepatitis C virus-associated hepatocellular carcinoma [27]. IL-17F was decreased in patients with pancreatic adenocarcinoma compared with chronic pancreatitis patients [39]. In bladder cancer, IL-17F was overexpressed in the cancer group compared with the cystitis and hyperplastic bladder polyp groups [11].

**Fig. 2:**
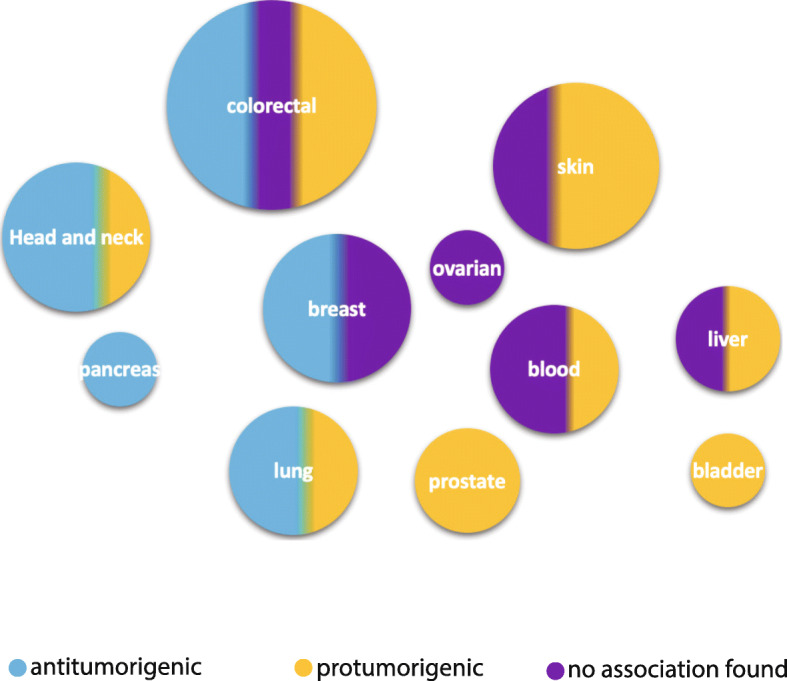
Interleukin-17F expression and its role in tumorigenesis. The size of dots represents the number of studies included in this review and the colour represents the possible role of IL-17F expression in tumorigenesis

To summarize, IL-17F expression (protein and mRNA) levels in tumour and serum samples seem to depend on cancer type (Fig. 2), and more studies are needed prior to concluding its predictive value in any of the malignancies.

### IL-17F SNPs

Eight IL-17F SNPs (rs763780, rs9382084, rs12203582, rs1266828, rs2397084, rs7771511, rs641701, and rs9463772) were studied in terms of their association with 13 types of cancer in 38 studies [17, 19, 42, 46–76]. Findings are collected in Tables 2 and 3. In six cancers – breast, cervical, laryngeal, liver, skin, and pancreatic cancer – no significant association was found with the IL-17F SNPs [42, 46–49, 52, 70, 71]. In six cancers – acute myeloid leukemia, bladder, colorectal, gastric, lung, and oral cancer – the results were inconsistent [17, 19, 50, 51, 53–69, 72–75, 77, 78]. One study reported significant association between rs763780 IL-17F SNP and high risk of developing follicular lymphoma [76].

**Table 2:** Summary of the studies concerning the role of IL-17F polymorphisms in cancer

SNP	Cancer type	Total studies No.	No association No.	Association No.
rs763780	Bladder	3	2	1
	Blood (AML)	3	2	1
	Breast	2	2	–
	Cervix	3	3	–
	Colorectal	7	4	3
	Gastric	10	7	3
	Oral	2	2	–
	Larynx	1	1	–
	Liver (HBV-HCC)	1	1	–
	Lung	2	1	1
	Lymphoma	1	–	1
	Pancreas	1	1	
	Skin (BCC)	1	1	–
rs9382084	Breast	1	1	–
	Cervix	1	1	–
	Gastric	1	–	1
rs12203582	Breast	1	1	–
	Gastric	1	1	–
	Lung	1	–	1
rs1266828	Breast	1	1	–
	Cervix	1	1	–
	Lung	1	1	–
rs2397084	Oral	2	1	1
	Lung	1	1	–
rs7771511	Breast	1	1	–
rs641701,	Colorectal	1	–	1
rs9463772	Colorectal	1	–	1

Abbrevations: AML = acute myeloid leukemia, BCC = basal cell carcinoma, HBV-HCC = hepatitis B virus-related hepatocellular carcinoma

**Table 3:** IL-17F polymorphisms in cancer

Cancer	SNPs	Population (country)	Study size (patients + healthy control)	Effect on patient (p-value)	Authors
Bladder	rs763780	Iran	180 + 180	The rs763780 polymorphism was not associated with bladder cancer susceptibility in the Iranian population.	Aslani et al., 2020 [72]
	rs763780	Poland	175 + 207	rs763780 polymorphism was not associated with bladder cancer susceptibility	Krajewski et al. 2020 [73]
	rs763780	China (Han)	301 + 446	TT genotype and T allele of rs763780 were more common among patients than controls. Rs763780 SNP was associated with bladder cancer development and tumor stage, as well as gender and smoking status of patient.	Zhou et al. 2013 [67]
Blood	rs763780	Egypt	100 + 100	IL-17F mutation showed neither correlation with AML susceptibility nor with therapy outcome.	Zayed et al. 2020 [74]
	rs763780	Egypt	100 + 100	IL-17F gene polymorphisms was not associated with AML risk	Elsissy et al. 2019 [50]
	rs763780	Poland	62 + 125	The rs763780 IL-17F polymorphism was found to be associated with predisposition to AML	Wróbel et al. 2014 [51]
Breast	rs763780	Southern Iran	192 + 215	No association between IL-17F polymorphisms and breast cancer susceptibility	Naeimi et al. 2014 [46]
	rs7771511, rs9382084, rs12203582, rs1266828, rs763780	China (Han)	491 + 502	No association between IL-17F polymorphisms and breast cancer susceptibility	Wang et al. 2012 [47]
Cervix	rs763780	China	352 + 352	No association between IL-17F polymorphisms and cervical cancer	Cong et al. 2015 [48]
	rs763780, rs9382084, rs1266828	China	264 + 264	No significant association between IL-17F polymorphisms and cancer risk	Lv et al. 2015 [49]
	rs763780	China	311 + 463	No association between IL-17F polymorphisms and cancer risk or patient clinical characteristics	Quan et al., 2012 [52]
Colorectal	rs641701, rs9463772	Italy	370 (test set *n* = 233, validation set *n* = 137)	rs641701 and rs9463772 were found to be a prognostic markers related to a high risk of LARC disease recurrence, metastasis, and death	Cecchin et al. 2020 [75]
	rs763780	China	352 + 433	IL-17F rs763780 polymorphism was not associated with the risk of CRC	Feng et al. 2019 [53]
	rs763780	Korea	695 + 1846	Dietary pattern reflecting inflammation was significantly associated with CRC risk. Moreover, this association could be modified according to the IL-17F rs763780 genotype and anatomic site.	Cho et al. 2018 [54]
	rs763780	Saudi Arabia	117 + 100	No association between IL-17F polymorphisms and CRC risk	Al Obeed et al. 2018 [55]
	rs763780	Malaysia	70 + 80	No association between IL-17F polymorphisms and CRC risk	Samiei et al. 2018 [56]
	rs763780	France	122	IL-17F polymorphisms was not associated with OS/PFS	Lereclus et al. 2017 [17]
	rs763780	Southern Iran	202 + 203	T allele of IL-17F T7488C may be involved in reduced risk of CRC	Nemati et al. 2015 [19]
	rs763780	Tunisia	102	IL-17F AG + GG genotypes were more frequent in controls than in patients with colon cancer, and IL-17F wild type genotype AA had an impact on OS.	Omrane et al. 2015 [57]
Gastric	rs763780	Korea	300 + 247	T allele frequency of IL-17F rs763780 was found to be statistically higher in patients with gastric cancer, compared with healthy controls	Choi et al. 2016 [58]
	rs763780	China	153 + 207	No association was found between IL-17F rs763780 T > C genotype and risk of gastric cancer	Zhao et al. 2016 [59]
	rs763780	China	326 + 326	No significant positive association was observed with the risk of gastric cancer and IL-17F polymorphisms	Hou et al. 2015 [60]
	rs763780	China	462 + 462	No significant differences between rs763780 genotypes and gastric cancer risk	Wang et al. 2014 [61]
	rs763780	Chile	147 + 172	IL-17F polymorphisms was not associated with risk of gastric cancer	Gonzalez-Hormazabali et al. 2014 [63]
	rs763780	China	572 + 572	rs763780 polymorphism may be associated with risk of developing gastric cancer, particularly among alcohol drinkers.	Gao et al. 2014
	rs763780, rs9382084, rs12203582	China	293 + 550	The rs9382084 TT genotype was significantly associated with an increased risk of gastric cancer and has interaction with tobacco smoking on gastric cancer risk. Rs9382084 genetic variants greatly increase risk of non-cardia gastric cancer. No association was found between variants of rs763780 and rs12203582 and gastric cancer risk	Qinghai et al. 2013 [64]
	rs763780	China (Han)	962 + 787	IL-17F polymorphisms associated with susceptibility to gastric cancer and clinopathological features of it	Wu et al. 2010 [65]
	rs763780	Japan	102	No significant association between CIHM status and IL-17F (7488 T > C)	Tahara et al. 2010 [66]
	rs763780	Japan	287 + 524	No significant difference between IL-17F genotypes and risk of gastric cancer	Shibata et al. 2009 [68]
Head and neck	rs763780, rs2397084	China	182 + 364	No association between rs763780 and rs2397084 polymorphisms and risk of oral cancer	Hu et al. 2017
	rs763780, rs2397084	China	121 + 103	Rs2397084, but not rs763780, was associated with OSCC risk, and this was related to tumor stage and differentiation. IL-17F polymorphisms together with smoking and drinking can enhance the risk of OSCC development	Li et al. 2015 [78]
Larynx	rs763780	China	325 + 325	IL-17F genotypes and alleles was not associated with risk of laryngeal cancer	Si et al. 2017 [70]
Liver	rs763780	China	155 + 171	IL-17F rs763780 polymorphisms do not contribute to HBV-related HCC susceptibility independently	Xi et al. 2015 [72]
Lung	rs763780, rs1266828, rs12203582	China	320 + 358	rs763780 and rs1266828 were not associated with lung cancer risk. Rs12203582 associated with risk of lung cancer, also among smokers.	He et al. 2017 [69]
	rs763780, rs2397084	Tunisia	239 + 258	IL-17F 7488G allele was associated with increased lung cancer risk. IL-17F7383 A/G polymorphism was not associated with lung cancer risk	Kaabachi et al. 2014 [62]
Lymphoma	rs763780	Brazil	152 + 212	rs763780 polymorphism increased risks of developing follicular lymphoma.	Assis-Mendonça et al., 2020 [76]
Pancreas	rs763780	European and African	351 (European ancestry *n* = 294, African ancestry *n* = 26)	OS was significantly shorter for the rs763780 heterozygotes compared with controls, but this did not hold in the multivariate analysis.	Innocenti et al. 2012 [71]
Skin	rs763780	Iran	200 + 200	No association of rs763780 gene polymorphisms and risk of BCC	Mohammadipour et al. 2019 [42]

Abbrevations: AML = acute myeloid leukemia, BCC = basal cell carcinoma, CIHM = CpG island hypermethylation, CRC = colorectal cancer, HBV = hepatitis B virus, HCC = hepatocellular carcinoma, LARC = locally Advanced Rectal Cancer, OS = overall survival, OSCC = oral squamous cell carcinoma, PFS = progression-free survival

### Proposed function of IL-17F based on in vivo and in vitro studies

We analysed the mechanisms by which IL-17F affects cancer development and progression based on in vitro and in vivo animal studies and collected the findings in Table 4.

**Table 4:** Interleukin-17F functional studies

Cancer	Study type	Cell lines/animal type	Main results	Authors
Blood	in vitro	PBMCs and CD4^+^ T cells	IL-17F triggers NFkB phosphorylation in T and B cells from patients with CLL, but not age-matched healthy controls.	Sherry et al. 2015 [24]
Breast	in vitro	MCF-7 cells	IL-17F enhances MCF-7 cell proliferation, migration and invasion via activation of the MAPK/ERK signaling pathway.	Chen et al. 2020 [81]
Colorectal	in vitro	HCT116 cells	IL-17F promotes cancer cell migration and invasion by inducing epithelial-mesenchymal transition	Chen et al., 2019 [15]
	in vitro	HCT116 wild-type and IL-17F overexpressing cell clones	IL-17F plays an important role in colon cancer development through regulation of cell cycle. This could partially happen through IL-17F effects on p27 and p38.	Tong et al. 2014 [82]
	in vitro, in vivo	Apc^Min/+^ mice, CRC cell lines (DLD-1 and HT-29)	Tumor-infiltrating leukocytes produce large amounts of T helper type IL17-related cytokines, including IL-17F. Individual neutralization of IL-17F does not change the TIL-derived proproliferative effect in CRC cells.	De Simone et al., 2014
	in vitro, in vivo	Cell lines (HCT116, HUVEC), BALB/c nude mice and C57BL/6 mice	IL-17F has protective role in colon cancer, possibly by inhibiting tumor angiogenesis.	Tong et al. 2012 [20]
Gastric	in vitro	Gastric cancer cell line (AGS)	IL-17F, may contribute to amplification and persistence of inflammatory processes implicated in inflammation-associated cancer through activation of p65 NFkB.	Zhou et al. 2007 [83]
Oral	in vitro	Cell lines (HSC-3, SCC-25, SAS)	IL-17F has an antitumorigenic effect through inhibition of the vasculogenic mimicry	Almahmoudi et al. 2021 [84]
	in vitro	Cell lines (HSC-3, SCC-25, HOKs, HUVEC, CAF)	IL-17F inhibited cell proliferation and random migration of oral cancer cells and inhibited the endothelial cell tube formation.	Almahmoudi et al. 2019 [21]
Liver	in vitro, in vivo	Cell lines (293 T, SMMC-7721, ECV304), athymic nude mice	IL-17F suppresses cancer cell growth via inhibition of tumor angiogenesis.	Xie et al. 2010 [85]
Lung	in vitro, in vivo	Human A549 and murine LL/2 (LLC1) lung cancer cell lines, bone marrow-derived macrophages from C57BL/6 mice. Chicken chorioallantoic membrane (CAM)	IL-17A/F does not affect cancer cell viability or glycolytic metabolism in vitro. Conditioned media from IL-17A/F-stimulated macrophages promoted lung cancer cell progression through an increased migration capacity in vitro and enhanced in vivo tumor growth, proliferation and angiogenesis.	Ferreira et al. 2020 [86]
	in vivo	CCSP^cre^/K-ras^G12D^ mice	IL-17F has no effect on lung cancer development in K-ras mutated mouse model.	Chang et al. 2014 [87]
Small intestine	in vivo	Apc^Min/+^ mice	Ablation of IL-17F significantly inhibits spontaneous intestinal tumorigenesis in the small intestine of ApcMin^/+^ mice. This was associated with decreased IL-1b and Cox-2 expression as well as IL-17 receptor C (IL-17RC) expression	Chae et al., 2011 [88]

Abbrevations: CLL = chronic lymphocytic leukemia, CAF = cancer-associated fibroblasts, HOKs = human oral keratinocytes, HUVEC = human umbilical vein endothelial cells, NFkB = Nuclear Factor kappa-light-chain-enhancer of activated B cells, PBMC = peripheral blood mononuclear cell, TIL = tumor-infiltrating leukocyte

#### Mechanisms for antitumour effects of IL-17F

Several mechanisms have been suggested through which IL-17F exert its antitumorigenic effects, including inhibition of tumour angiogenesis, cell cycle regulation, cancer cell proliferation and migration, and cancer vasculogenic mimicry. IL-17F inhibited tumour angiogenesis in three cancer types: liver [85], colon [20], and oral [21, 84]. IL-17F inhibited oral carcinoma cell proliferation, random cell migration and vasculogenic mimicry [21, 84], controlled the cell cycle through p27 and p38, and diminished oxidative stress-causing G2/M phase arrest in colon carcinoma cells [82].

#### Mechanisms for protumorigenic effects of IL-17F

In addition to the possible macrophage-mediated, pro-angiogenic role of IL-17F presented by Ferreira et al. [86], IL-17F was found to contain also other protumorigenic properties, through inflammation [83], epithelial-mesenchymal transition [15] NFkB regulation [24] and MAPK/ERK activation [81]. Zhou et al. [83] showed that IL-17F might enhance inflammatory processes in inflammation-associated cancer through activation of p65 NFkB. Similarly, Sherry et al. [24] reported that IL-17F causes NFkB phosphorylation in T- and B-cells of CLC patients.

Conditioned media from IL-17F-stimulated macrophages promoted lung cancer progression by enhancing cancer cell migration in vitro and tumour growth in vivo [86], but no connection existed between IL-17F and lung tumour number in a mouse model [87], nor was there any connection between lung cancer cell viability and glycolytic metabolism in vitro [86].

The CRC cell migration and invasion promoting role of IL-17F was caused by induction of epithelial-mesenchymal transition of HCT-116 cells [15]. Similarly, Chen et al. [81] found that IL-17F enhances MCF-7, a breast cancer cell line, cell proliferation, migration and invasion via activation of the MAPK/ERK signaling pathway. In Apc^Min/+^ mice, knockout of IL-17F inhibited small intestine tumorigenesis, and this was associated with decreased IL-1b, Cox-2, and IL-17RC expression [88]. In addition, IL-17F was not associated with a TIL-derived proliferative effect in CRC [89].

## Discussion

In this systematic review, we aimed to clarify the role of IL-17F, protein and mRNA expression and polymorphisms, in cancer and the mechanisms through which IL-17F affects cancer development and progression. We collected publications from four databases (Ovid Medline, PubMed, Scopus, and Cochrane Library). Based on the collected data, IL-17F seems to play a role in cancer development and progression. However, this role was shown to be either pro- or antitumorigenic depending on the cancer type, the source (tissue or fluid) from which it was measured, and the form (protein or mRNA) in which it was analyzed. The correlation between IL-17F polymorphism and cancer incidence or patients’ prognosis seemed to be weak in every cancer analyzed. Effects of IL-17F on cancer progression were shown to be through several different mechanisms.

The correlation between IL-17F expression and cancer has been studied in 13 different cancers in 34 publications. The results have varied in different cancers and depending on the expression (protein or mRNA) and location (tumour tissue or soluble) of IL-17F. The main findings concerning IL-17F protein expression were in OSCC, which showed that IL-17F expression in tumor tissue and patient serum, but not in saliva, was associated with better prognosis [22, 23, 44, 45]. The opposite effect was noticed in prostate cancer [40, 41]. In the case of colorectal cancer and lymphomas, which were studied quite extensively, the results were inconclusive [15–20, 31–36, 43]. In other cancers, there were only a few studies or the results varied too much to draw a clear conclusion. This variation in the role of IL-17F in different cancers is common also in other proteins such as MMP-8 [90]. Despite IL-17A and IL-17F binding to the same receptors, namely IL-17RA and IL-17RC, and sharing high homology, IL-17A has a clearer protumorigenic effect [9], while the role of IL-17F is variable. One clear example of the variation between the two cytokines is their role in oral cancer. As mentioned earlier, IL-17F was shown to be an antitumour cytokine in most of the analysed articles in this review, while IL-17A was reported more than once to be a protumour cytokine [91, 92].

The association between IL-17F polymorphisms and cancer was another important aspect in this review, but again the results were variable. Rs763780 polymorphisms have been broadly studied in CRC and gastric cancers [17, 19, 53–61, 63–66, 68, 75], but the findings have been diverse. Other IL-17F SNPs has been less extensively analyzed, and more studies are needed to confirm the relevance of IL-17F polymorphisms in various cancers. As with the expression results, the data for IL-17A polymorphisms are more solid than for IL-17F. The results of a meta-analysis covering 10 case-control studies, involving 4516 cases and 5645 controls, showed a significant association between IL-17A polymorphisms and the risk of developing cancer, particularly gastric cancer, in the Asian (and Chinese) population [93].

There were only a few functional studies of IL-17F, suggesting both an anti- and protumorigenic roles. One of the main mechanisms by which IL-17F exerts its antitumorigenic effect is the inhibition of angiogenesis and vasculogenic mimicry, which was reported in four studies [20, 21, 84, 85]. Inhibition of tumour angiogenesis and vasculogenic mimicry could thus be a potential therapeutic target in cancer treatment [94]. Other antitumorigenic mechanisms of IL-17F included the inhibition of cancer cell proliferation and migration [21] and cell cycle regulation [82]. A protumorigenic role of IL-17F could be caused by regulation of inflammatory responses [83], epithelial-mesenchymal transition [15], IL-1b, Cox-2, and IL-17CR expression [88] and MAPK/ERK activation [81]. Since Ferreira et al. [86] assessed the effects of IL-17A and IL-17F simultaneously, their findings cannot be extrapolated to IL-17F alone. In our search for the functional studies of IL-17F in cancer, we detected two interesting articles reporting opposite results about the role of IL-17F in colon cancer. Tong and his colleagues claimed an anti-tumorigenic role for IL-17F by showing a significant decrease in the tumor growth when IL-17F over expressed HCT116 cells transplanted subcutaneously in nude mice comparing with the mock transfectants [20]. They also used AOM-DSS induced inflammation-associated colon cancer IL-17F^−/−^ mice model to show that these mice had higher colonic tumor numbers and tumor areas compared with the wild-type controls. After further analysis, they found that this anti-tumorgenic role in both models was possibly a result of inhibition of tumor angiogenesis through decreasing VEGF levels and CD31^+^ cells. On the other hand, Chae and Bothwell used Apc^Min/+^ mice model highly susceptible to develop spontaneous intestinal adenoma to study the effect of IL-17F on intestinal cancer [88]. Opposite to the Tong et al., IL-17F knockout Apc^Min/+^ mice inhibited the spontaneous intestinal tumorigenesis compared with the Apc^Min/+^ mice. This was also associated with reducing IL-1β, Cox-2, and IL-17RC expression suggesting proinflammatory and protumorgenic roles of IL-17F in intestinal cancer [88]. These contrary results are confusing, however they could be due to the different mice models used in the two studies.

In contrast to IL-17A, which has already been studied intensively, IL-17F has thus far received much less attention in the cancer research field. Based on our criteria, only 79 articles were included in this review, which is a low number considering that we included all types of cancer. Weaknesses of this review comprise its broad scope and the large number of different types of studies, making it impossible to provide an in-depth analysis of them. Additionally, studies included in this article examined a variety of different designs, study populations and primary/secondary outcomes, which unfortunately reduce the value of direct interstudy comparisons and therefore these comparisons should be taken with caution. Nevertheless, this review gives an overall picture of the variable IL-17F roles in different cancers.

In conclusion, more well-designed studies of IL-17F are needed to elucidate its molecular mechanisms in different types of cancer. These studies are important to address several aspects such as the difference in the function between tissue or soluble IL-17F, the affected pathways through which IL-17F exert its effect, the effect of IL-17F on the tumor stroma cells, especially inflammatory cells, and if targeting IL-17F could provide a therapeutic benefits for cancer patients.

## Data Availability

The datasets supporting the conclusions of this article are included within the article.

## Abbreviations

BIA-ALCL: breast implant-associated anaplastic large cell lymphoma
CLL: chronic lymphocytic leukaemia
CRC: colorectal cancer
CTCL: cutaneous T-cell lymphoma
IL: interleukin
MAPK/ERK: mitogen-activated protein kinase/ extracellular signal-regulated kinases
OTSCC: oral tongue squamous cell carcinoma
PMNs: polymorphonuclear leukocytes
PRISMA: Systematic Reviews and Meta-Analyses
